# Author Correction: An Early Cretaceous pterosaur with an unusual mandibular crest from China and a potential novel feeding strategy

**DOI:** 10.1038/s41598-020-70506-z

**Published:** 2020-08-11

**Authors:** Xiaolin Wang, Taissa Rodrigues, Shunxing Jiang, Xin Cheng, Alexander W. A. Kellner

**Affiliations:** 1 Key Laboratory of Vertebrate Evolution and Human Origins of Chinese Academy of Sciences, Institute of Vertebrate Paleontology and Paleoanthropology, Chinese Academy of Sciences, P. O. Box 643, Beijing, 100044 China; 2 Department of Biology, Agrarian Sciences Center, Universidade Federal do Espírito Santo, Alto Universitário s/n, Caixa Postal 16, Guararema, CEP 29500–000 Alegre, ES Brazil; 3 University of Chinese Academy of Sciences, Beijing, 100049 China; 4 Laboratory of Systematics and Taphonomy of Fossil Vertebrates, Department of Geology and Paleontology, Museu Nacional/Universidade Federal do Rio de Janeiro, Quinta da Boa Vista s/n, São Cristóvão, CEP 20940–040 Rio de Janeiro, RJ Brazil

Correction to: *Scientific Reports*10.1038/srep06329, published online 11 September 2014

This Article Wang et al. (2014), which was published electronically, does not include evidence of registration in ZooBank within the work itself, as required by Art. 8.5.3 of the amended Code of the International Commission on Zoological Nomenclature (ICZN, 1999)^1^. Therefore, the newly proposed genus-group name *Ikrandraco* and species-group name *I. avatar* are not available from that work. This issue is addressed with this Correction.

This publication has been registered in ZooBank with the LSID: urn:lsid:zoobank.org:pub:60C039F2-4505-44C7-85EA-92306D626D7B. Below, we give the ZooBank LSID numbers for each of the two new names, along with the Systematic Palaeontology section of the original article^2^.

**Systematic Paleontology**

Pterosauria Kaup, 1834

Pterodactyloidea Plieninger, 1901

Dsungaripteroidea Young, 1964

Pteranodontoidea Marsh, 1876

*Ikrandraco* gen. nov.

urn:lsid:zoobank.org:act:E5A43C54-E155-47D4-8515-5510A9F24B69

**Type species**

*Ikrandraco avatar*, type by monotypy.

**Etymology**

*Ikran*, from the fictional flying creature portrayed in the movie Avatar that shows a well developed dentary crest and *draco*, from the Latin meaning dragon.

**Diagnosis**

The same for the type species.

*Ikrandraco avatar* sp. nov.

urn:lsid:zoobank.org:act:E7C1E2F4-6D50-441B-B30C-9C6DED9C2276

**Etymology**

Avatar, in allusion to the homonymous science fiction movie.

**Holotype**

A partial skeleton including complete skull and mandible, atlas, axis, three mid-cervical vertebrae, part of the sternal plate, some ribs, partial right and left wings and part of the foot (IVPP V18199), deposited at the Institute of Vertebrate Paleontology and Paleoanthropology, Chinese Academy of Sciences in Beijing, China.

**Referred specimen**

A complete skull, mandible, atlas, axis and third cervical vertebra (IVPP V18406), deposited at the Institute of Vertebrate Paleontology and Paleoanthropology, Chinese Academy of Sciences in Beijing, China.

**Horizon and locality**

Jiufotang Formation, Early Cretaceous (Aptian) 120 Ma. IVPP V18199 was collected in Lamadong, Jianchang and IVPP V18406 comes from Sihedang, Lingyuan, western Liaoning, China.

**Diagnosis**

*Ikrandraco avatar* can be distinguished from all other pteranodontoid pterodactyloids by the following autapomorphies: slightly arched dorsal margin of the skull above the nasoantorbital fenestrae; lateral depression on the nasal; median hook-like process on the posterior edge of the dentary crest; two well-developed pneumatic foramina piercing the lateral surface of the axis. It can further be separated from other pteranodontoids by the following combination of characters: very low skull (height over quadrates about 18.7% of skull length); strongly inclined quadrate (150°); lack of a premaxillary crest; presence of a deep, blade-like bony mandibular crest with deepest point at mid-length; and a ventral pneumatic foramen on the proximal portion of the second phalanx and third phalanges of the wing finger.
